# Analysis of non-ventriculoperitoneal shunts at Red Cross War Memorial Children’s Hospital

**DOI:** 10.1007/s00381-023-06242-2

**Published:** 2023-12-13

**Authors:** B. G. De John, A. A. Figaji, J. M. N. Enslin

**Affiliations:** 1 Department of Surgery, Division of Neurosurgery, University of Cape Town, Cape Town, South Africa; 2 Division of Neurosurgery, Red Cross War Memorial Children’s Hospital, Cape Town, South Africa

**Keywords:** Hydrocephalus, Ventriculoatrial, Ventriculopleural, Cerebrospinal fluid diversion

## Abstract

**Background:**

At Red Cross War Memorial Children’s Hospital (RCCH), it is the preferred practice to use non-ventriculoperitoneal (non-VP) shunts when the peritoneum is ineffective or contraindicated for cerebrospinal fluid (CSF) diversion and when endoscopy is not an option. The objective of this study is to evaluate the clinical course of patients having undergone these procedures.

**Method:**

A single-centre retrospective review at RCCH wherein 43 children with a total of 59 episodes of non-VP shunt placement over a 12-year period were identified for inclusion.

**Results:**

Twenty-five ventriculoatrial (VA) and 32 ventriculopleural (VPL) shunts were analysed with a median age at insertion of 2.9 (0.3–14.9) and 5.3 years (0.5–13.4), respectively. The median number of previous shunt procedures prior to VA or VPL shunt insertion was 6.0 (2–28) versus 4.5 (2–17), respectively. Three VA (12.0%) and three VPL (9.4%) shunt patients were lost to follow-up. Of those remaining, 10 VA shunts (45.5%) compared to 19 (65,5%) VPL shunts required revision. One ventriculovesical shunt and one ventriculocholecystic shunt were placed in the same patient after 21 and 25 shunt-related procedures, respectively, and both were revised within 3 weeks of insertion. Median shunt survival was 8 months longer for the VA compared to the VPL shunts, being 13.5 (0–67) and 5 months (0–118), respectively. Complications for VA shunts were low, with the overall shunt sepsis rate in the VA group at 4% (*n* = 1) compared to 15.6% (*n* = 5) in the VPL group.

**Conclusion:**

Our findings support that VA and VPL shunts are acceptable second-line options in an already compromised group of patients where safe treatment options are limited, provided attention is paid to the technical details specific to their placement.

## Introduction

Hydrocephalus (HCP) causes death and disability by increased pressure due to cranial accumulation of cerebrospinal fluid (CSF) [1]. It represents a major public health concern globally: Dewan et al. estimated nearly 400,000 new cases of paediatric hydrocephalus annually worldwide, 180,000 of which occur in Africa [2, 3].

Historically, CSF diversion procedures have been met with varying degrees of success and 36 diversion sites have been reported [4–6]. First attempts by Ferguson in 1898 involved CSF drainage from the lumbar theca to the peritoneum with a silver wire. In 1908, Payr introduced shunting from the ventricle directly into the sagittal sinus and jugular veins, whereas Kausch utilised a rubber conduit and drained CSF into the peritoneal cavity [7, 8]. Heile, in 1914, drained CSF to the pleural space and later in 1925 developed the first ventriculoureteral shunt [8]. In 1952, the first implantable ventriculoatrial (VA) shunt system was developed by Frank Nulsen and Eugen Spitz [4, 5, 7–9]. With the invention of silicone catheters and one-way valves by John Holter in 1956, these shunt systems could better withstand long-term mechanical stresses [9]. Following this, insertion of these systems via the venous route became popular, predominantly into the right atrium. Ransohoff in 1954 returned to the practice of ventriculopleural (VPL) shunting, but this was limited by the development of pleural effusions and shunt obstruction [8, 10].

By the late 1960s, Ames, Raimondi, and Matsumoto popularised the ventriculoperitoneal (VP) shunt technique, utilising the improved silicone devices, and by the 1970s, VA shunts became infrequent. This was largely due to the ease of VP shunt insertion and revision rather than long-term efficacy [5, 8, 10, 11]. VP shunts, additionally, required fewer revisions following anatomical growth and had less potential for severe complications [5, 9, 11, 12]. Consequently, at most centres, modern neurosurgeons have much less experience with placement of non-peritoneal shunts.

At Red Cross War Memorial Children’s Hospital (RCCH), it has been the preferred practice to use non-VP shunts when the peritoneum has been deemed ineffective or is contraindicated for CSF diversion and where endoscopic third ventriculostomy (ETV) is not an option or has failed. Relative contraindications to peritoneal placement include peritonitis, pancreatitis, ascites, traumatic abdominal injuries, and significant adhesions following previous abdominal pathology or surgery [4]. At out institution, VPL shunts were the first of the second-line options. Hoffman et al. and Jones et al. reported on their use of VPL shunts, supporting them as a safe alternative with better tolerance and fewer problematic pleural effusions in children over the age of 4 years [10, 13]. Over time, however, we have raised concerns about VPL shunt survival in our setting due to the high pulmonary tuberculosis (PTB) rate, reported to be 737 per 100,000 population [14]. Other concerns include the perceived high complication and poor survival rates of these systems in our context. We therefore have migrated towards VA shunt placement in this group of patients.

The objective of this study is to evaluate our current practice and outcomes of second-line CSF shunt placement when VP shunt placement or endoscopy is not possible or feasible. We aimed to compare this to the literature and historical context of paediatric VA and VPL shunting.

## Methodology

### Study population

This is a single-centre retrospective review at the RCCH Neurosurgical Division, Cape Town, South Africa. All children under the age of 13 years (exceptions to this discussed below) who underwent non-VP shunt placement at RCCH between the 1st of January 2009 and the 31st December 2020 were included. Patients were identified from prospectively maintained databases, and their medical and operative records were reviewed. We excluded patients in whom a non-VP shunt had been inserted at another facility.

### Shunt technique

Most VA shunts were inserted by one of the authors (JE), utilising a percutaneous, ultrasound-guided, Seldinger technique with fluoroscopy to guide shunt placement. Where this was not possible, a formal open cut-down procedure was performed. More recently, however, distal catheter insertion length was calculated by measuring the distance from the planned skin incision to the sternal angle (angle of Louis) in order to limit radiation exposure and improve procedural workflow. This, together with chest lead electrocardiogram (ECG) monitoring, aimed to ensure catheter placement in the distal third of the superior vena cava. Catheter selection was typically an antibiotic-impregnated proximal and distal catheter with a medium pressure Medtronic Atlas^®^ valve.

Placement site for the VPL shunts was surgeon-dependent, with incision at the right-sided 5th intercostal space in the mid-axillary line being the preferred practice. Surgeon allocation was less specific. Blunt dissection to the pleura was followed by pleural opening and insertion of a shortened distal catheter, under Valsalva manoeuvre.

Post-operative chest X-rays were routinely utilised in both VA and VPL shunt procedures to confirm catheter position and to exclude complications.

There were only single attempts at ventriculocholecystic (VC) and ventriculovesical (VV) shunts, both of which occurred in the same patient after all other avenues were exhausted. These were performed as an open surgical procedure using an antibiotic-impregnated catheter with the assistance of paediatric surgery and urology teams, respectively.

### Clinical and surgical variables

Variables collected included the following: demographic information (age, sex, timing, and number of CSF diversion procedures); aetiology of hydrocephalus; comorbid conditions; evidence of previous tuberculosis infection; indication for non-VP shunt insertion; procedure-related factors; surgical outcomes (time to shunt failure; reason for failure; immediate-, short- and long-term complications; immediate revision procedure following failure); and time of follow-up.

### Definitions

Typically, children are only managed until 12 years of age at RCCH, after which they are transferred to the adult division at another hospital. Exceptions to this are patients with significant comorbidities and small habitus who warrant ongoing treatment by a paediatric multidisciplinary team until the age of 18 years at RCCH. We divided complications into immediate, short and long term. Immediate complications occurred at the time of surgery, short-term complications within 30 days of surgery, and long-term complications after 30 days from surgery. Patients were “lost to follow-up” if they did not attend their 3-month follow-up appointment.

### Data analysis

Data was described by measures of central tendency, and univariate and multivariate analysis was conducted through Intel SPSS software^®^.

### Ethical considerations

Ethical approval was granted by the University of Cape Town Human Research Ethics Committee (REF: 317/2020). All data was anonymised and ethical considerations were maintained throughout.

## Results

Between 2009 and 2020, a total of 44 eligible patients were identified. A single patient was excluded because of insertion of a VPL shunt at another institution, leaving 43 patients with non-VP shunts and 59 episodes of non-VP shunt insertion procedures amongst them.

### Baseline characteristics

A total of 25 VA shunts (42.4%), 32 VPL shunts (54.2%), one VV shunt (1.7%), and one VC (1.7%) shunt were inserted in those 43 patients. Twenty-five patients were male and 18 female. Nine patients (56.3%) in the VA shunt group and 12 patients (44.4%) in the VPL group had multiple medical comorbidities (see Table 1). Most hydrocephalus cases were of unknown aetiology (15 patients (34.9%)), followed by myelomeningocele in nine patients (20.9%), post-infectious hydrocephalus in five patients (11.6%), and tuberculous hydrocephalus in four patients (9.3%). The patient who underwent both the VV and VC shunt procedures had post-infectious hydrocephalus.

**Table 1:** Demographics and baseline characteristics

		**Ventriculoatrial**		**Ventriculopleural**		**Ventriculovesical**		**Ventriculocholecystic**		**Total**	
		*n*	%	*n*	%	*n*	%	*n*	%	*n*	%
Route of non-VPS procedure		25	42.4%	32	54.2%	1	1.7%	1	1.7%	59	100.0%
Sex	Male	9	56.3%	16	59.3%					25	58.1%
	Female	7	43.8%	11	40.7%					18	41.9%
Median age in years at first CSF diversion (range)		0.4	(0–1.5)	0.6	(0–5.0)	0.8		0.8		0.6	(0–5.0)
Mean age in years at first CSF diversion (± SD)		0.5	(0.4)	1.0	(1.2)	0.8		0.8		0.8	(1.0)
Median age in years at non-VP shunt (range)		2.9	(0.3–14.9)	5.3	(0.5–13.4)	3.6		3.8		4.0	(0.3–14.9)
Mean age in years at non-VP shunt (± SD)		5.2	(4.6)	5.9	(4.1)	3.6		3.8		5.5	(4.3)
Median number of previous shunt procedures (range)		6.0	(2–28)	4.5	(2–17)	21.0		25.0		5.0	(2–28)
Mean number of previous shunt procedures (± SD)		7.7	(6.2)	6.2	(4.4)	21.0		25.0		7.4	(6.0)
Multiple medical comorbidities	No	7	43.8%	15	55.6%	1	100.0%	1	100.0%	22	51.2%
	Yes	9	56.3%	12	44.4%					21	48.8%
Aetiology of HCP	Idiopathic	6	37.5%	9	33.3%					15	34.9%
	Myelomeningocele	5	31.3%	4	14.8%					9	20.9%
	Post-infectious	1	6.3%	4	14.8%	1	100.0%	1	100.0%	5	11.6%
	4th ventricular outflow obstruction	2	12.5%	3	11.1%					5	11.6%
	TBM			4	14.8%					4	9.3%
	Intraventricular haemorrhage	1	6.3%	2	7.4%					3	7.0%
	Tumour related			1	3.7%					1	2.3%
	Aqueductal stenosis	1	6.3%							1	2.3%
	Total	16	100.0%	27	100.0%	1	100.0%	1	100.0%	43	100.0%

### Preceding treatment

The median age at first CSF diversion procedure (VP shunt) was 0.4 years (0–1.5 years) for VA shunts versus 0.6 years (0–5.0 years) for VPL shunts. The median age at insertion of a VA shunt was 2.9 years (0.3–14.9 years) and 5.3 years (0.5–13.4 years) for VPL shunts. The median number of previous shunt procedures prior to VA shunt insertion was 6.0 (2–28) versus 4.5 (2–17) for VPL shunts. VV and VC shunts were placed in the same patient after 21 and 25 shunt-related procedures, respectively. Most indications for non-VPS insertion were related to abdominal pathology (see Table 2) with 32.2% of shunts inserted due to the presence of abdominal pseudocysts (suspected low-grade infection), 22% due to proven intra-abdominal sepsis, 11.9% due to abdominal adhesions and CSF malabsorption, 10.2% for hollow viscus perforations/erosions, and 3.4% for iatrogenic bowel injury. One (1.7%) non-VP shunt was inserted due to abdominal tuberculosis. The remaining indications included multiple failed VP shunts with concern of peritoneal malabsorption of CSF (1.7%) or failed other non-VP shunts and persistence of the above-mentioned scenarios (17.0%).

**Table 2:** Indication for non-ventriculoperitoneal shunt insertion

		**Ventriculoatrial**		**Ventriculopleural**		**Ventriculovesical**		**Ventriculocholecystic**		**Total**	
		*n*	%	*n*	%	*n*	%	*n*	%	*n*	%
Indication non-VPS	Abdominal pseudocyst	8	32.0%	11	34.4%					19	32.2%
	Intra-abdominal sepsis	6	24.0%	7	21.9%					13	22.0%
	Significant abdominal adhesions	3	12.0%	2	6.3%	1	100.0%	1	100.0%	7	11.9%
	Bowel/bladder perforation/erosion	2	8.0%	4	12.5%					6	10.2%
	Abdominal surgery	2	8.0%	3	9.4%					5	8.5%
	VPL shunt dysfunction	1	4.0%	3	9.4%	1	100.0%			5	8.5%
	Pleural effusion related to VPL shunt	3	12.0%							3	5.1%
	VA shunt dysfunction	2	8.0%	1	3.1%					3	5.1%
	Multiple failed VPS (concern of peritoneal malabsorption)	1	4.0%							1	1.7%
	Bowel injury	1	4.0%	1	3.1%					2	3.4%
	TB abdomen			1	3.1%					1	1.7%
	Vent-vesical shunt dysfunction							1	100.0%	1	1.7%
	Vent-GB shunt dysfunction	1	4.0%							1	1.7%

### Shunt survival and failure

#### Follow-up time

Of the 25 VA shunts, three (12.0%) were lost to follow-up (patients from other provinces). In the VPL group, three (9.4%) were lost to follow-up. The median time of follow-up was 1.9 years (0–8.5 years) for the VA shunt group and 4.2 years (0.3–10.0 years) for the VPL group.

#### Revision rate

Of the 22 VA shunt patients with follow-up, 10 (45.5%) required revision of their VA shunt (see Table 3). Nineteen VPL patients (65.5%) required revision. Both VV and VC shunts were revised within 3 weeks of placing them due to malabsorption of CSF and hydrocephalus.

**Table 3:** Shunt survival and reason for failure

		**Ventriculoatrial**		**Ventriculopleural**		**Ventriculovesical**		**Ventriculocholecystic**		**Total**	
		*n*	%	*n*	%	*n*	%	*n*	%	*n*	%
Route of non-VPS procedure		25	42.4%	32	54.2%	1	1.7%	1	1.7%	59	
Lost to follow-up		3	12.0%	3	9.4%					6	10.2%
Revision of non-VPS (Y/N)	No	12	54.5%	10	34.5%					22	37.3%
	Yes	10	45.5%	19	65.5%	1	100.0%	1	100.0%	31	52.5%
Median follow-up time in years		1.9	(0–8.5)	4.2	(0.3–10.0)					2.7	(0–10.0)
Mean follow-up time in years (± SD)		2.6	(2.3)	4.4	(3.0)					3.5	(2.8)
Median shunt survival time in months (range)		13.5	(0–67)	5	(0–118)					6.0	(0–118)
Mean shunt survival time in months (± SD)		19.3	(21.1)	21.9	(33.5)					20.0	(28.3)
Shunt survival at	3 months	16	72.7%	18	62.1%					34	100.0%
	6 months	13	59.1%	14	48.3%					27	79.4%
	12 months	11	50.0%	10	34.5%					21	61.8%
	18 months	11	50.0%	9	31.0%					20	58.8%
Reason shunt failure	Shunt blockage	6	60.0%	5	26.3%	1	100.0%	1	100.0%	13	41.9%
	Pleural effusion			7	36.8%					7	22.6%
	Shunt sepsis			4	21.1%					4	12.9%
	Displaced distal catheter	1	10.0%	3	15.8%					4	12.9%
	Shunt disconnection	1	10.0%	1	5.3%					2	6.5%
	Intracranial sepsis			1	5.3%					1	3.2%
	Endocarditis	1	10.0%							1	3.2%
	Shunt nephritis	1	10.0%							1	3.2%
	Unsuccessful shunt insert	1	10.0%							1	3.2%
	Pleural empyema			1	5.3%					1	3.2%
Immediate revision procedure	External ventricular drain	3	30.0%	9	47.4%	1	100.0%	1	100.0%	14	45.2%
	Ventriculoperitoneal shunt	6	60.0%	6	31.6%					12	38.7%
	Ventriculopleural shunt	1	10.0%	3	15.8%					4	12.9%
	Ventriculovesical shunt			1	5.3%					1	3.2%
	Total	10	100.0%	19	100.0%	1	100.0%	1	100.0%	31	100.0%

#### Shunt survival

**Fig. 1:**
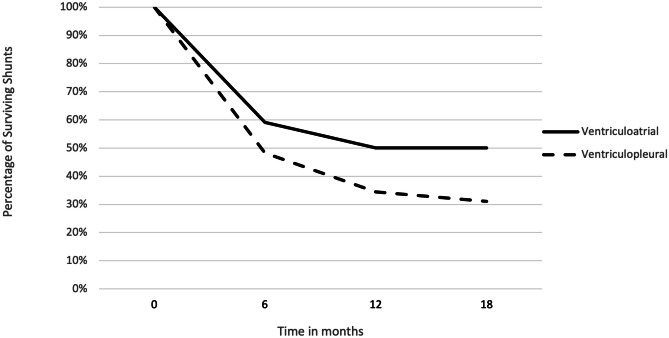
Non-ventriculoperitoneal shunt survival over time (as percentage)

**Fig. 2:**
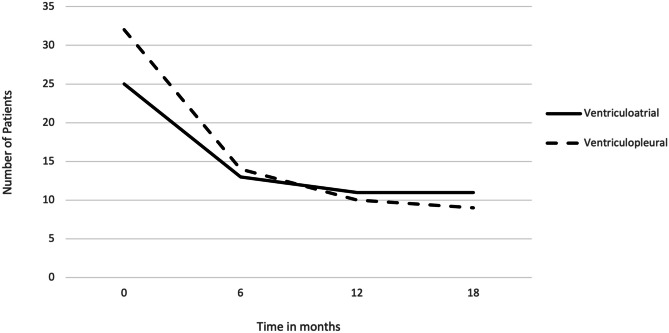
Non-ventriculoperitoneal shunt survival over time (absolute count)

Median shunt survival for the VA shunts was 13.5 months (range 0–67) and 5 months (range 0–118) for VPL shunts. Of the 22 VA shunts included, survival rates at 3, 6, 12, and 18 months were 72.7%, 59.1%, 50.0%, and 50.0%, respectively. In comparison, shunt survival in the 29 VPL shunts was 62.1%, 48.3%, 34.5%, and 31.0% at 3, 6, 12, and 18 months, respectively (see Figs. 1 and 2). The VV shunt and VC shunt survived 4 and 21 days, respectively, before shunt malfunction became evident.

In the failed VA shunts, shunt blockage accounted for six cases (60.0%). Other causes included one shunt disconnection (10.0%), one displacement of the distal catheter (10.0%), and one endocarditis with shunt nephritis (10.0%). Unsuccessful VA shunt insertion was noted in one case (10.0%) necessitating a VPL shunt insertion at the time of the attempted VA shunt surgery. Shunt failure in the VPL group was due to shunt blockage in five cases (26.3%), symptomatic pleural effusions in seven (36.8%), shunt sepsis in three (15.8%), and displacement of the distal catheter in three (15.8%). There was one case (5.3%) of pleural empyema.

In cases requiring revision of their VA or VPL shunt, the immediate revision procedure was a VP shunt or EVD in nine (90.0%) and 15 cases (79.0%), respectively. In the VA and VPL shunt revision group, there was sustained management of HCP with VP shunts in five (50.0%) and 11 cases (57.9%), respectively. The median time to initial revision of the VA and VPL shunts in this group was 75 and 157 days, respectively. Fifty percent of VA (*n* = 5) and 42.1% (*n* = 8) of VPL shunt revision cases required ongoing management of HCP with non-VP shunts. The median time to initial revision in this group is 34 days and 16 days, respectively.

### Complications

#### Immediate complications

These were more common in the VA shunt group with nine events in total (see Table 4). These were minor in nature and included non-significant, transient arrhythmia (*n* = 3), deep atrial insertion (*n* = 1), failed Seldinger technique requiring formal open cut down (*n* = 3), and arterial puncture (*n* = 1). In the VPL group, there was one event of surgical emphysema that required no further intervention.

**Table 4:** Complications of non-ventriculoperitoneal shunts

		**Ventriculoatrial**		**Ventriculopleural**	
		*n*	% of total	*n*	% of total
Immediate surgical complications	Non-significant arrythmia	3	12.0%		
	Failed Seldinger technique	3	12.0%		
	Deep atrial insertion	1	4.0%		
	Arterial puncture	1	4.0%		
	Minor blood loss	1	4.0%		
	Surgical emphysema			1	3.1%
	Total	9	36.0%	1	3.1%
Short-term complications (30 days)	Asymptomatic pleural effusion			6	18.8%
	Symptomatic pleural effusion			5	15.6%
	Pneumothorax (no intervention)			2	6.3%
	Displaced distal catheter			2	6.3%
	Ventriculitis			1	3.1%
	Subdural hygroma	1	4.0%	1	3.1%
	Deep catheter in the atrium	2	8.0%		
	Superficial wound infection	1	4.0%		
	Total	4	16.0%	17	53.1%
Long-term complications	Shunt sepsis			3	9.4%
	Symptomatic pleural effusion			2	6.3%
	Asymptomatic pleural effusion			1	3.1%
	Empyema			1	3.1%
	Displaced distal catheter			1	3.1%
	Death			1	3.1%
	Subdural hygroma	1	4.0%	2	6.3%
	Shunt disconnection	1	4.0%	1	3.1%
	Endocarditis	1	4.0%		
	Shunt nephritis	1	4.0%		
	Total	4	16.0%	12	37.5%

#### Short-term complications

These were more common in the VPL group and included symptomatic pleural effusion in five cases (15.6%) requiring shunt revision, asymptomatic pleural effusion in six cases (18.8%) requiring no intervention, and one shunt sepsis (3.1%). The VA shunt group had two cases of deep atrial insertion of the distal catheter on post-op screening (8.0%) and no episodes of shunt sepsis.

#### Long-term complications

These were more common in the VPL group with 10 events in total, including two symptomatic pleural effusions, one pleural empyema, and three shunt sepsis events, one of which resulted in death. The death occurred in a palliative patient known with severe baseline disability who represented with shunt sepsis and demised shortly thereafter, prior to any further intervention from neurosurgery. In the VA shunt group, the long-term complications were fewer but significant, with one event of endocarditis with shunt nephritis which required VA shunt revision after appropriate temporary diversion and intravenous antibiotics. Of note, the patient made a good recovery. Overall, the shunt sepsis rate in the VA shunt group was 4% (*n* = 1) and 15.6% (*n* = 5) in the VPL group.

### Operative technique

Ultrasound assistance (Seldinger technique) was used in 92.0% (*n* = 23) of the VA shunt insertions, with 16.0% (*n* = 4) of the shunts requiring a formal open neck dissection (see Table 5). Intra-operative fluoroscopy was performed in 28.0% (*n* = 7). Most cases (92.0%, *n* = 23) received post-operative antibiotics for at least 24 h.

**Table 5:** Surgical-related factors

		**Ventriculoatrial**		**Ventriculopleural**		**Ventriculovesical**		**Ventriculocholecystic**		**Total**	
		*n*	%	*n*	%	*n*	%	*n*	%	*n*	%
Brand of shunt used	OSVII	5	20.0%	2	6.3%					7	11.9%
	Bactiseal	16	64.0%	17	53.1%	1	100.0%	1	100.0%	35	59.3%
	Miethke	1	4.0%							1	1.7%
	Not stated	3	12.0%	11	34.4%					14	23.7%
	Essential			2	6.3%					2	3.4%
Ultrasound-guided technique (Y/N)	No	2	8.0%							2	3.4%
	Yes	23	92.0%							23	39.0%
	N/A			32	100.0%	1	100.0%	1	100.0%	34	57.6%
Formal open cut down	No	21	84.0%							21	35.6%
	Yes	4	16.0%							4	6.8%
	N/A			32	100.0%	1	100.0%	1	100.0%	34	57.6%
X-ray screening intra-op	No	18	72.0%							18	30.5%
	Yes	7	28.0%							7	11.9%
	N/A			32	100.0%	1	100.0%	1	100.0%	34	57.6%
Antibiotics post-op	Yes	23	92.0%	24	75.0%	1	100.0%	1	100.0%	49	84.5%
	Not stated	2	8.0%	8	25.0%					9	15.5%

## Discussion

VP shunts remain the preferred CSF diversion procedure to manage hydrocephalus, with the use of non-VP shunts reserved for instances in which the peritoneum is contraindicated or has failed previously, except where ETV is appropriate [15, 16].

### Efficacy of VA and VPL shunts

The Shunt Design Trial and Hydrocephalus Research Network’s studies demonstrate a shunt failure rate of up to 47% in the first 2 years [17, 18]. By comparison, the VA shunt failure rate of 45% (median follow-up 1.9 years) in our current series is not dissimilar. However, the VPL shunt group had a 62% failure rate (median follow-up 4.2 years). Although the duration of follow-up for the latter group is longer, there appears to be a difference at 12 months (Fig. 1). Although differences between the groups due to the non-randomised selection are unavoidable, it is notable that the VA shunted patients were younger and therefore a higher rate of shunt dysfunction may have been expected.

The lower shunt survival rate for VA and VPL shunts is expected as these procedures are typically performed as second-line measures within a very heterogenous subset of patients who already have had several shunt complications. The patient demographic (low- and middle-income) and spectrum of disease may contribute to this. Warf et al. reported that up to 60% of hydrocephalus in Africa is due to infection [19]. In our study, 9% of hydrocephalus was related to myelomeningocele, 11.6% to post-infectious cases, and 9.3% to TBM-related hydrocephalus. It is likely that many of the unknown aetiology group were post-infectious cases. Additionally, the insertion of VA and VPL shunts is undertaken in complex patients as evidenced by the high rate (48.8%) of medical comorbidities and the high number of previous shunt-related procedures in our sample. One child had 28 prior shunt procedures.

Direct comparison between VPL and VA shunts as a second-line option is sparse and is compromised by small numbers and relatively short follow-up and compounded by the greater complexity of these patients in recent series [20]. Much of the available literature derives from historical series where these systems were inserted as a primary procedure and may not be directly applicable because surgical and perioperative techniques have changed over the intervening years. These earlier studies, however, highlight the efficacy of these systems in the absence of other compounding comorbidities. Keucher and Mealey in 1979 demonstrated similar mortality and infection rates for VA and VP shunts in 228 patients with infantile non-neoplastic hydrocephalus, although VA shunts had more revisions, and late complications were more frequent and severe [12].

Vernet et al., from 1970 to 1991, reviewed 120 cases of infantile hydrocephalus who underwent VA shunting as a primary procedure [21]. With an average follow-up of 11 years, they demonstrated no operative mortality and only one shunt-related death which was secondary to shunt nephritis. Their infection rate was 4.2% with an average revision rate of 2.2 per patient. Of note, 66% of revisions were for elective lengthening of the atrial catheter [21]. Due to this disadvantage, they supported VP shunts over VA shunts as a primary procedure [21]. A Norwegian study of 128 children followed up children who received a VA shunt as a primary procedure between 1967 and 1970 over a 45-year period: 30% of shunts were revised within a year, and 73% within the first decade, with 26.3% of revisions done for elective lengthening of the catheter [22]. Rymarczuk et al. showed no difference in the survival of VA vs VP shunts, excluding elective lengthening procedures in the VA group [23].

Of interest in the adult population with VA shunts, where fewer revisions due to growth are needed, Lam and Villemure (49 patients) and Al-Schameri et al. (255 patients) demonstrated similar infection and complication rates between VP and VA shunts [24, 25]. Both favoured VP shunts as a primary procedure due to ease of placement and less potential for a severe complication [24].

Yavuz et al. studied VA shunts as a second-line option in 10 patients aged 5 to 13 years. They reported three revisions due to thrombosis, endocarditis, and pulmonary embolus [26]. Clark et al. also studied 94 VA shunt insertions in 38 patients as a second-line intervention. They reported higher revision rates to ours, with shunt survival rates of 53%, 43%, and 27% at 6, 12, and 24 months, respectively, and an overall infection rate of 11% [27]. They concluded that the percutaneous ultrasound-guided technique was safe with a serious adverse event rate of only 2%.

We prefer to reserve the use of VPL shunts for children over the age of 4 years, due to concerns about pleural effusions where lung capacity and compliance may be reduced. Hoffman et al. had a similar approach; 12 (20%) of their 59 patients developed pleural effusions, six of which were under 11 months of age. Twenty-three of their patients required no revision [10]. Jones et al., in 52 VPL shunt patients (mean age of 8 years), reported three shunt infections, four catheter obstructions, one symptomatic pleural effusion, and one death from shunt malfunction [13]. Martínez-Lage et al., in six patients (5 to 13 years), noted no revisions after a mean follow-up of 2.5 years [28]. In an adult population, where pleural effusion may be less concerning, Craven et al. in 2017 studied 22 VPL shunts and reported a median shunt survival of 14 months [29].

In a recent review, Forte et al. found similar results to ours in their VPL and VA shunt comparison [20]. In a series of 36 VA shunt and 18 VPL shunt insertions over 15 years, VA shunt survival was 60.6%, 51.5%, and 36.4% at 3, 6, and 12 months, respectively, while VPL shunt survival was 56.3%, 43.8%, and 37.5%, respectively [20]. Median time to shunt revision was 8.5 and 5.5 months for VA and VPL shunts, respectively. We concur with their conclusions about the role of VA or VPL shunts as a second-line procedure. They advised consideration of VA over VPL shunt insertion in those under 5 years [20]. Rymarczuk et al. in their review of 85 VA shunt patients over a 13-year period further agree with the second-line role these shunt systems serve and demonstrated similar outcomes to those above [23].

VA and VPL shunt use may afford time for the peritoneum to heal, allowing later re-introduction of a VP shunt. In our series, VP shunting was undertaken for 50.0% of VA (*n* = 5) and 57.9% (*n* = 11) of VPL shunt revisions. Once the original insult contraindicating the peritoneum has resolved, a VP shunt can be re-considered in the settings of non-VPS failure.

### Current technique of VA shunts

With our shifting focus from VPL to VA shunts, one of the objectives was to evaluate the VA shunt placement technique. The Seldinger technique (percutaneous guidewire assisted placement), first described in 1981, has become preferred [30]. Clark et al. described the assistance of ultrasound guidance and intra-operative fluoroscopy to confirm distal tip position [27]. More recently, Della Pepa et al. reported venous catheter insertion under ultrasound guidance with ECG-guided distal tip positioning [31]. This technique utilises an electrode-integrated venous catheter and relies on predictable changes in the ECG p-wave trace as the atrium is approached [31]. This technique was similarly described by Muhammad et al. and by McCracken et al. [32, 33].

In our study, 23 (92.0%) VA shunts were inserted with the Seldinger technique under ultrasound guidance, with intra-operative fluoroscopy performed in seven (28,0%). We noted that a technique using patient measurements together with chest lead ECG monitoring, to aid in correct catheter placement within the lower third of the superior vena cava, is safe and effective. Deep IVC placement was seen in only two cases (neither of these cases were done with fluoroscopy nor measurement to the angle of Louis techniques, with rather an estimate of 10 cm used instead by the surgeon); both these patients remained well and have not required revision as of 2022.

### Complications

Short- and long-term complications were more common in the VPL group, most of which (*n* = 7/21.9%) were related to pleural effusions with one case of pleural empyema (3.1%). This compared to the cohort by Forte et al., which reported a rate of pleural effusions at 22.2% [20]. In our study, the shunt sepsis rate for the VA and VPL shunt group was 4% (*n* = 1) and 15.6% (*n* = 5), respectively, compared to infection rates reported by Forte et al., of 13.8% and 5.6% for VA and VPL shunts [20].

Reported complications for VPL shunts include pneumothorax, lung injury, ventilatory difficulties, pneumocephalus, tension hydrothorax, and fibrothorax [4]. Small asymptomatic pleural effusions are also commonly described [34]. Reported VA shunt complications include catheter thrombosis, thrombo-embolism (including pulmonary emboli), vessel perforation, bacterial endocarditis, arrythmia, nephritis, pulmonary hypertension, and cor pulmonale [4, 11, 12, 35]. Interestingly, Vandersteene et al. demonstrated a pro-coagulant effect of CSF which is attributable to coagulation proteins and tissue factor [36]. Generally, CSF concentrations in the venous system are well below the critical threshold required; however, in certain circumstances, they may increase the risk of clot formation [36]. Shunt nephritis was first described in 1965 and is typically thought to arise from infection with low virulence organisms, triggering an immune complex deposition at the glomerular basement membrane [11, 35, 37]. In our one case of shunt nephritis, a VP shunt was inserted after antibiotic treatment. At the time of this study, this patient is still doing well, with no signs of recurrence and no long-term sequelae.

Limitations of this study include its retrospective nature, descriptive methodology, limited long-term follow-up, and the small cohort, the size of which precluded a reliable comparative statistical analysis. Due to the more recent insertion of VA shunts, a lower median follow-up time was encountered, which may also contribute to the apparent higher survival rate of these shunt systems. Comparison to conventional VP shunt survival and complications is limited by the selection criteria. Larger studies with longer term follow-up are recommended in order to establish robust clinical guidelines. However, due to small numbers, a multicentre approach is necessary.

## Conclusion

Our findings are comparable to recent studies of similar design and support the use of VA and VPL shunts as effective second-line CSF diversion procedures. Additionally, we have, at times, found these procedures to act as a useful interim measure, “buying time” until the peritoneum can be re-considered as a CSF diversion site. No shunt operation is without its risks, but it is clear that in an already compromised group of patients where safe treatment options are limited, VA and VPL shunts remain good alternative options and should not be discarded by the neurosurgeon. Trainees should be taught correct, safe surgical techniques to reduce complications.

## Data Availability

No datasets were generated or analysed during the current study.

## Abbreviations

CSF: Cerebrospinal fluid
ECG: Electrocardiogram
ETV: Endoscopic third ventriculostomy
EVD: External ventricular drain
HCP: Hydrocephalus
PTB: Pulmonary tuberculosis
RCCH: Red Cross War Memorial Children’s Hospital
TBM: TB meningitis
VA shunt: Ventriculoatrial shunt
VC shunt: Ventriculocholecystic
VP shunt: Ventriculoperitoneal shunt
VPL shunt: Ventriculopleural shunt
VV shunt: Ventriculovesical shunt
